# The Development and Testing of RELOAD-C, a Web-Based Platform to Reduce Loneliness in Dementia Caregivers

**DOI:** 10.1093/geroni/igaf122.719

**Published:** 2025-12-31

**Authors:** Allison Marziliano, Allison Applebaum, Edith Burns, Marzena Gieniusz, Jessica Mongelli, Huma Babar, Liron Sinvani, Michael Diefenbach

**Affiliations:** Northwell Health, New Hyde Park, New York, United States; Icahn School of Medicine at Mount Sinai, New York, New York, United States; Zucker School of Medicine Hofstra-Northwell, Manhasset, New York, United States; Northwell Health, New Hyde Park, New York, United States; Northwell Health, New Hyde Park, New York, United States; Northwell Health, New Hyde Park, New York, United States; Northwell Health, New Hyde Park, New York, United States; Northwell, Manhasset, New York, United States

## Abstract

There is a need for evidence-based interventions to reduce loneliness in family caregivers of patients with dementia. Given the inverse relationship between finding meaning in life and loneliness, interventions to reduce loneliness may be strengthened by incorporating concepts from Meaning-Centered Psychotherapy (MCP), a psychotherapy effective in increasing meaning in life in patients with advanced cancer and their caregivers. The purpose of this presentation is to describe the development and preliminary testing of RELOAD-C (REducing LOneliness in Alzeheimer’s Disease-Caregivers), a web-based platform that delivers concepts from MCP, adapted for caregivers of patients with dementia, via 6 brief videos, 7 virtual peer group meetings, and written content. During development, N = 15 dementia caregivers completed one-on-one interviews with the study PI. Input from stakeholders indicated: 1) preference for specific terminology; 2) removing MCP content that urges caregivers to engage in complex conversations with the person they care for; 3) providing an explanation of ambiguous concepts, such as “unfinished business”; and 4) refraining from using the term “intervention” given its negative connotations. Following integration of stakeholder feedback and creation of the RELOAD-C web-based intervention, usability/acceptability testing occurred with a separate 16 caregivers. Of the 16 caregivers, n = 13 (81.3%) correctly completed all 10 discreet tasks. Averages on 9/10 items of the System Usability Scale were below 2.0 (possible range 1-5), indicating favorable usability of RELOAD-C. In sum, RELOAD-C is a user-friendly intervention, informed by multiple rounds of review by a multidisciplinary research team and stakeholders from the very population it is designed to help.

